# Emergence of isolates that are intrinsically resistant to colistin in critically ill patients: are we selecting them out?

**DOI:** 10.1186/cc14175

**Published:** 2015-03-16

**Authors:** MN Sivakumar, M Hisham, V Nandakumar, T Gopinathan

**Affiliations:** 1 Kovai Medical Center and Hospital, Coimbatore, India

## Introduction

Poor infection control practices along with irrational usage of antibiotics lead to emergence of multidrug-resistant (MDR) organisms. Increasing use of colistin for treating MDR infections leads to selection of organisms that are intrinsically resistant to colistin. There are limited Indian literatures which evaluated the incidence of intrinsically resistant isolates to colistin in critically ill patients. Our study aimed to investigate the incidence of true pathogen or colonizer with the prior antibiotic exposure and patient's clinical outcome.

## Methods

The prospective, cross-sectional study was carried out from March 2013 to April 2014. Clinical samples included culture positivity for isolates intrinsically resistant to colistin from patients who were admitted to the ICU or had a prior ICU stay in the same admission.

## Results

A total of 93 unusual Gram-negative rods were isolated from 76 patients. This included 19.4% (*n *= 18) *Serratia marcescens*, 12.9% (*n *= 12) *Stenotrophomonas maltophilia*, 14% (*n *= 13) *Burkholderia cepacia*, 24.7% (*n *= 23) *Proteus mirabilis*, 17.2% (*n *= 16) *Morganella morganii*, 9.7% (*n *= 9) *Elizebethkingia meningoseptica *and 2.1% (*n *= 2) *Providencia *species. A total of 68.4% (*n *= 52) patients had prior exposure to either colistin or carbapenems or both. In total, 71% (*n *= 66) of the total isolates from patients had previous antibiotic exposure. Among the total 93 intrinsically resistant isolates to colistin, 37.6% (*n *= 35) of isolates from all clinical sources (endotracheal, pus, urine and blood samples) were true pathogens and the remaining 62.3% (*n *= 58) were colonizers. There was a statistically significant increase in length of ICU stay and duration of hospitalization in the presence of true pathogen.

## Conclusion

Selection pressure due to extensive use of higher antibiotics may lead to emergence of intrinsically resistant isolates, which narrows the therapeutic options in the ICU. Our study emphasizes the paramount importance of establishing clinical relevance of these organisms before treating them as true pathogens. This calls for judicious use of higher antibiotics, implementation of an antibiotic stewardship program and strict infection control practices.
